# Molecular Simulations of Low-Shrinkage Dental Resins Containing Methacryl-Based Polyhedral Oligomeric Silsesquioxane (POSS)

**DOI:** 10.3390/polym15020432

**Published:** 2023-01-13

**Authors:** Chandra Mouli R. Madhuranthakam, Sudharsan Pandiyan, Ali Elkamel

**Affiliations:** 1 Chemical Engineering Department, Abu Dhabi University, Abu Dhabi 59911, United Arab Emirates; 2 Schrodinger India Private Limited, Bengaluru 560 098, Karnataka, India; 3 Chemical Engineering Department, University of Waterloo, Waterloo, ON N2L 3G5, Canada; 4 Chemical Engineering Department, Khalifa University, Abu Dhabi 127788, United Arab Emirates

**Keywords:** methacrylate POSS, molecular dynamics simulations, elastic modulus, dental resins, powder diffraction, nanocomposite

## Abstract

Nanocomposites of methacrylate-based polyhedral oligomeric silsesquioxane (POSS) are used as resins in dentistry to fill dental cavities. In this article, molecular dynamics simulations (MDS) are used to study and understand the interactions of monofunctional and multifunctional methacrylate groups on hybrid resins containing POSS additives for dental applications. These interactions are further related to the structural properties of the nanocomposites, which in turn affect their macro-properties that are important, especially when used for specific uses such as dental resins. For monofunctional methacrylate, nanocomposite of methacryl isobutyl POSS (MIPOSS) and for multifunctional methacrylate, methacryl POSS (MAPOSS) are used in this study. Molecular dynamic simulations (MDS) are performed on both MIPOSS and MAPOSS systems by varying the amount of POSS. On a weight percent basis, 1%, 3%, 5%, and 10% POSS are added to the resin. Density calculations, stress–strain, and powder diffraction simulations are used to evaluate the macro-properties of these nanocomposites and compare them with the experimental findings reported in the literature. The observations from the simulation results when compared to the experimental results show that MDS can be efficiently used to design, analyze, and simulate new nanocomposites of POSS.

## 1. Introduction

Search for novel polymeric composites with high mechanical stability and higher curing rate is underway for dental applications. Specific polymers named “dental resins” are used for cavity-filling applications. Adding hole-filling agents such as polyhedral oligomeric silsesquioxanes (POSS) to these polymers was found to improve the properties of the resins for dental applications. The experimental efforts have already been devoted to understanding the influence of POSS on the material properties of the resins. However, molecular simulations would be an ideal tool for understanding their properties at the molecular level. This insight into the molecular level relationship between the structure of additives and the resin will lead to the design of better novel materials. Polyhedral oligomeric silsesquioxanes (POSS) also called “spherosiloxanes” due to their spherical topology are a class of three-dimensional oligoromeric organosiliceous compounds. They have cage frameworks with different degrees of symmetry. They are 1–3 nm in diameter with the inner core made up of silicon and oxygen while the outer side is bonded to an organic functional group (R) leading to (RSiO_1.5_) bond units. These organic functional groups in POSS can be reactive or nonreactive which makes them useful in a wide range of applications. With the nonreactive R, they become soluble and compatible with other polymers, while with reactive R, they are amenable for use in grafting and polymerization reactions. Further, when R constitutes only one reactive group (also called monofunctional POSS), POSS can be incorporated as a pendant in a network while if R has more than two reactive groups (also called multifunctional POSS), POSS can be used as a crosslinking point. The inorganic core of POSS improves rigidity and stability while the organic functional groups affect flexibility, ductility, and processability when mixed with other materials. POSS with specific mono and multifunctional reactive groups are used as modifiers and crosslinkers in several polymer matrices targeted for improved mechanical, viscoelastic, barrier, and thermal properties. POSS has been a primary choice for synthesizing high-performance materials as it combines the benefits of organic and inorganic material characteristics. POSS is widely used in plastic additives, fluorinated superhydrophobic coatings, nanofiber membranes, electronics and energy applications, and biomedical applications [1,2,3,4,5]. Among biomedical applications, methacryl POSS, methacryl isobutyl POSS, and methacrylethyl POSS are used as new-generation dental composites [6,7,8].

Among the several works related to the study of the effect of POSS on dental composites, Wang et al. [9] reported on how the compatibility, photocuring behavior, morphology, mechanical and shrinking properties are affected by the different functionalized POSS structures of the organic substituent on the inorganic cage. Their study was mainly on two functionalized POSS, methacryl isobutyl POSS (MIPOSS) with only one methacrylate functional group and methacryl POSS (MAPOSS) that has eight methacrylate functional groups. It was experimentally shown that MAPOSS outperforms MIPOSS with respect to flexural modulus, flexural strength, wear resistance, and volume shrinkage which is attributed to their hypothesis of well-separated POSS being uniformly distributed into bisphenol A glycerolate dimethacrylate (Bis-GMA) and tri(ethylene glycol) dimethacrylate (TEGDMA) dental composite resins. The aim of this research was to simulate the dental composites used by Wang et al. and further analyze the corresponding composite properties using molecular dynamic simulations (MDS). By confirming the experimental findings with the theoretical simulations, it helps to use MDS not only to troubleshoot and tailor existing nanocomposites but also to design new composites, which would reduce the time and cost invested in experimental research. MDS is a powerful tool used to perform atomistic simulations of the behavior of new or existing materials [10,11]. Several macro properties of materials, such as elastic modulus, glass transition temperature, barrier properties, diffusion coefficient, and free volume can be theoretically obtained from analysis using MDS [12,13]. Very scant and specific literature is available on using MDS on epoxy systems with POSS (such as norbornene-POSS [14], polyamide-POSS [15], polyvinylidene fluoride-POSS [16], polyethyleneglycol-POSS [17] and polyethylene oxide-POSS [18]). In this article, different weight percent of MAPOSS and MIPOSS are mixed with the control which consists of bisphenol A glycerolate dimethacrylate (Bis-GMA) and tri(ethylene glycol) dimethacrylate (TEGDMA). These nanocomposite resins are crosslinked, and MD simulations are performed to obtain their properties which in turn are compared to the experimental findings reported by Wang et al. [9].

## 2. Simulations Details

**Figure 1:**
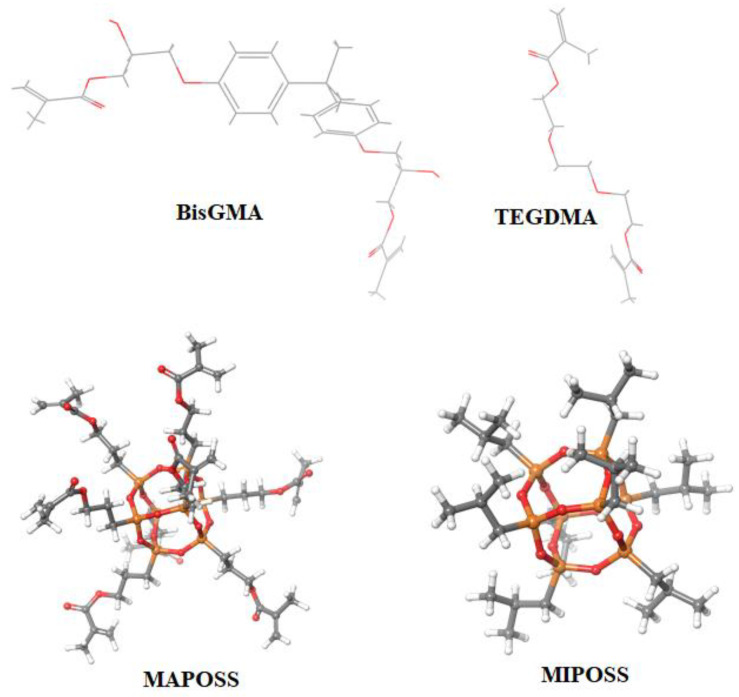
Chemical structures of BisGMA, TEGDMA, MAPOSS, and MIPOSS.

All molecular dynamics simulations were performed using the Materials Science (MS) Suite version 4.8.134 of Schrödinger 2022-4 release (Schrödinger, LLC, New York, NY, USA), which employed the OPLS4 force field [19]. Using the 2D and 3D sketchers in MS Maestro, the chemical structures of BisGMA, TEGDMA, MAPOSS, and MIPOSS are drawn as shown in Figure 1. Using the Disordered System builder within the framework of MS Suite desired composite structures are made, which is followed by material relaxation that consists of 20 ps NVT Brownian minimization at 10 K, a 20 ps NPT Brownian minimization at 100 K, a 100 ps NPT molecular dynamics at 300 K, and finally, 10 ns molecular dynamics at 300 K and 1.01325 bar. Crosslinking of the resultant structures is done after the material relaxation and equilibration. With 299 molecules of BisGMA and 381 molecules of TEGDMA, a crosslinked control is obtained (using an NVT ensemble at 600 K) that corresponds to 50 wt% of each of those polymers. This is referred to as “Control” in the subsequent portion of this article. Crosslinked composites with 1 wt%, 3 wt%, 5 wt%, and 10 wt% of MAPOSS and MIPOSS are obtained by using the same procedure. All crosslinked structures are relaxed for 50 ns using an NPT ensemble at 300 K and 1.01325 bar. The exact number of molecules of the individual components in the nanocomposite and their corresponding weight percentages are provided in Table 1.

The stress–strain calculations were performed using the option of volume being conserved in all three directions, with a Poisson ratio of 0.5, a strain rate of 0.04 × 10^8^ s^−1^, and using a strain step size of 0.001 for 200 steps. These simulations are run for a maximum strain of 0.16, and the Young’s modulus is estimated from the slope of the linear portion within the elastic limit of the stress–strain curve. The simulation protocol involved using a simulation time of 250 ps with a time step of 2.0 fs and trajectory recording interval of 10 ps at a temperature of 300 K. Powder diffraction simulations are performed for all crosslinked composites using CuKa radiation with a wavelength of 1.54184 Å with a 2θ range between 5° and 70°.

## 3. Results and Discussion

**Figure 2:**
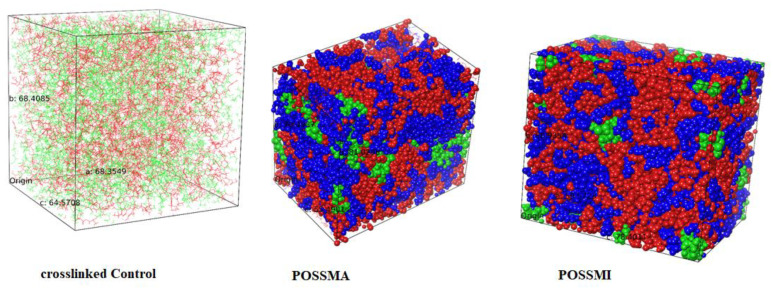
Equilibrated Control (BisGMA-green chains, TEGDMA—red chanins), POSSMA5 and POSSMI5 systems (blue spheres—BisGMA, red spheres—TEGDMA, green spheres—POSS).

**Figure 3:**
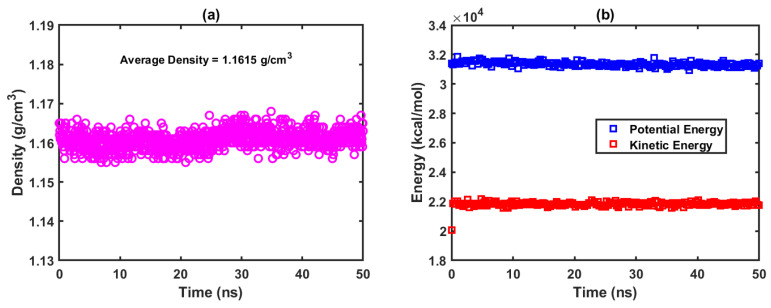
(**a**) Density vs. time profile; (**b**) cohesive energy vs. time profile for the Control.

The densities of the crosslinked control and other composites specified in Table 1 are obtained from the compressive relaxation of these systems for 50 ns. Figure 2 shows relaxed crosslinked molecular systems of the “Control”, POSSMA and POSSMI composites (with 5 wt% of corresponding POSS). Figure 3a shows the variation of density with respect to the simulation time for the “Control” while Figure 3b shows the corresponding profile obtained for the potential and kinetic energies. Figure 3 clearly shows that a well-equilibrated system is achieved from the converged density and energy profiles within the production run of 50 ns. Similar profiles are obtained for all crosslinked composites with MAPOSS and MIPOSS.

**Figure 4:**
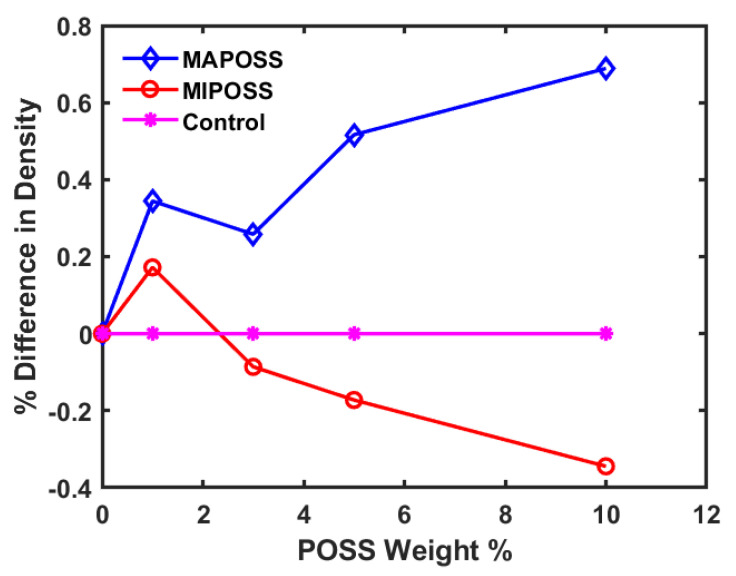
Comparison of density difference as a percentage difference for MAPOSS and MIPOSS composites as a function of POSS composition in weight %.

With respect to the density differences, the density of MAPOSS composites increased slightly as the amount of MAPOSS in the composite increased, while an opposite trend was observed with MIPOSS (see Figure 4). Methacrylate-based resins undergo a reduction in the free volume, which is due to a decrease in the distance between the atom groups that involve in a covalent bond resulting due to radical polymerization of C = C. This results in the shrinkage of the polymer which can be calculated using Equation (1).

(1) $$\left(\frac{\Delta V}{V}\right)\%=\frac{\rho_{cured}-\rho_{uncured}}{\rho_{cured}}\times 100\%$$

where (ΔV/*V*)% is the volume % shrinkage, *ρ_cured_* and *ρ_uncured_* are the densities of cured and uncured polymer, respectively. For most of the resins used as dental composites, volume shrinkage is usually in the range of 1 to 6% [20]. Wang et al. [9] reported a range of 2.8 to 3.6% and 2.5 to 3.4% for MAPOSS and MIPOSS, respectively. In our study, the volume shrinkage obtained was 3.35 ± 0.04% and 3.20 ± 0.03% for MAPOSS and MIPOSS, respectively. These values are in complete agreement with the range of experimental values reported. The small deviation in volume shrinkage values obtained from simulations can be due to the difference in operating temperature and also the high crosslinking conversion achieved in the simulations. The experimental conversion was reported between 70 and 80%, while in the simulations, the conversion was observed to be over 95%. This is acceptable in simulations as it is easy to find a molecule for crosslink within the small simulation volume compared to the actual volume in experiments. The experimental values are reported at temperatures 20 °C to 22 °C while the simulation results correspond to 27 °C. With an increase in temperature, the volume shrinkage of dental resin usually increases [21,22]. From both the reported experimental and our simulation results, it was observed that MAPOSS has more volume shrinkage compared to MIPOSS.

**Figure 5:**
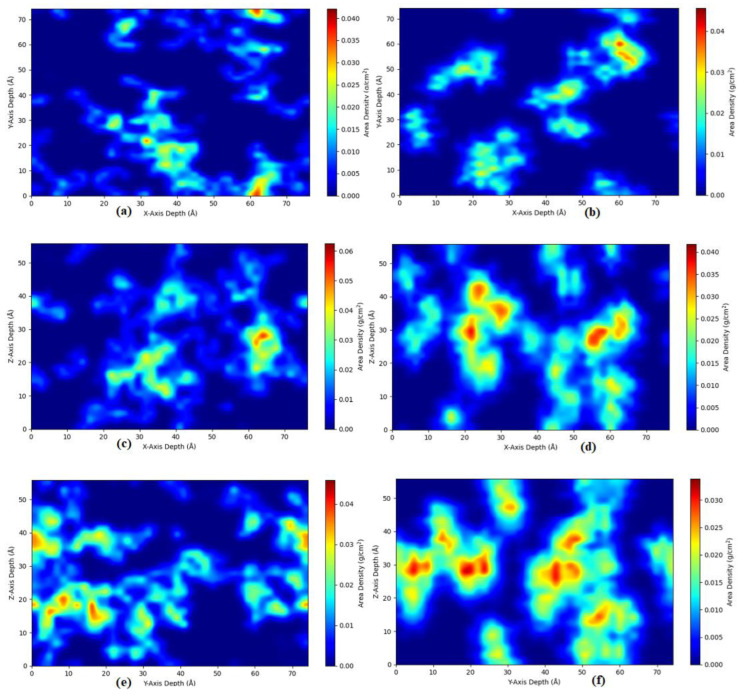
POSS density projection plot (**a**,**c**,**e**)—MAPOSS10, (**b**,**d**,**f**)—MIPOSS10.

**Figure 6:**
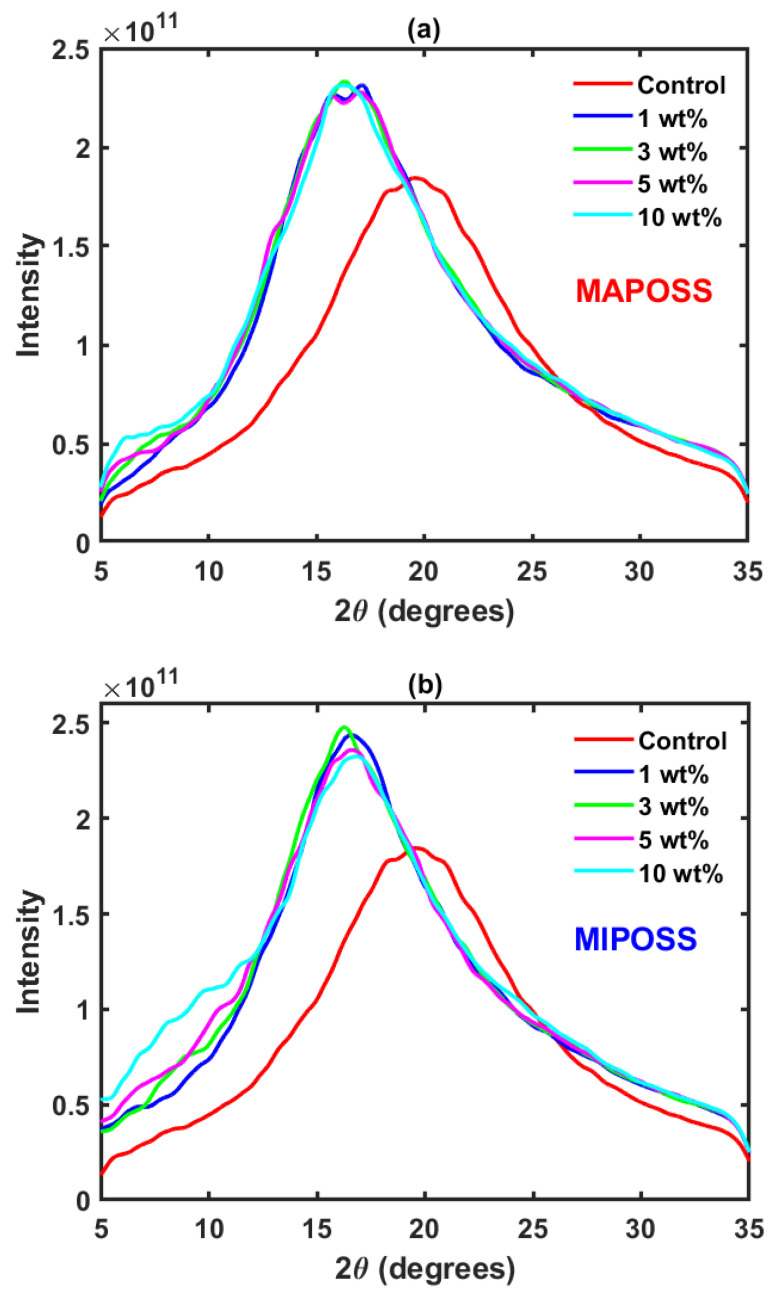
Powder diffraction profiles for (**a**) MAPOSS and (**b**) MIPOSS composites.

MAPOSS has double bonds which are also involved in the curing/crosslinking process which in turn makes it bonded to the molecules of the polymer matrix while MIPOSS does not have double bonds which essentially makes it a standalone filler. Further, MIPOSS forms agglomerates within the polymer matrix while MAPOSS is more uniformly distributed in the polymer matrix. This is clearly observed as shown in Figure 5 which shows the two-dimensional density projections XY, YZ, and ZX for MAPOSS10 (a, c, e) and for MIPOSS10 (b, d, f). The images shown in Figure 5 are obtained over 1000 frames during the 50 ns production run. In Figure 5a,c,e, MAPOSS is not moving at all, evidenced by the uniform blue color and the low agglomeration shown in non-blue regions. But MIPOSS shows mobility and also high-intensity agglomeration as shown in Figure 5b,d,f. This is in complete agreement with the experimental observations shown by Wang et al. [9] using SEM and TEM images. This observation is also supported by the results obtained using the powder diffraction simulations for all POSS composites. The results obtained for the intensity versus 2θ for MAPOSS and MIPOSS composites are shown in Figure 6.

From Figure 6a, the intensity profiles with respect to 2θ for all MAPOSS composites resemble the Control profile with two shoulders at 2θ = 6° and 2θ = 35°. From Figure 6a,b, for both MAPOSS and MIPOSS composites, the magnitude of the main peaks is greater than that of the corresponding peak for Control. The position of the broad peak which is characteristic of amorphous nature in both MAPOSS and MIPOSS shifts to 2θ = 16.7° compared to that of Control which occurs at 2θ = 19.8°. This small lateral shift is common in polymer composites with POSS [22,23]. For MIPOSS composites (as shown in Figure 6b, compared to the Control, the shoulder at 2θ = 6° is not observed. Even with a 1 wt% addition of MIPOSS, the intensity profile broadens with respect to Control in the region 6 < 2θ < 13 and the magnitude of the deviation increase with an increase in the amount of MIPOSS added to the composite. This can be attributed to the characteristic crystalline peaks within this region. This is in complete agreement with Wang et al. [9] who reported crystalline peaks appearing at 2θ = 7.3°, 8.0°, 10.0°, 11.76°, and 18.8° from the wide-angle X-ray diffraction results. In MIPOSS composites, MIPOSS molecules aggregate into microcrystals whose number and size increase with the MIPOSS loading.

**Figure 7:**
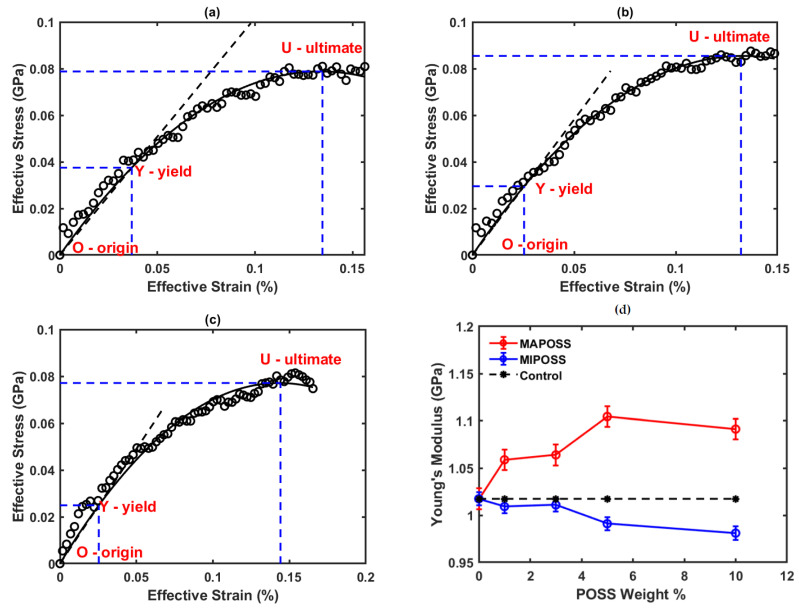
Effective stress vs. strain for (**a**) Control (**b**) MAPOSS10 and (**c**) MIPOSS10, (**d**) Young’s modulus vs. POSS wt% for all composites.

The Young’s modulus is estimated from the slope of the stress–strain curve within the elastic limit as shown in Figure 7a–c for Control, MAPOSS10, and MIPOSS10, respectively. The Young’s modulus of all other composites specified in Table 1 was estimated using a similar approach.

For more details on the graphical analysis of the stress–strain data for obtaining Young’s modulus, elastic stress, elastic strain, ultimate stress, and ultimate strain, refer to Madhuranthakam et al. [12,13]. The major focus of our study in this article is the evaluation of Young’s modulus for different weight fractions of POSS used in the nanocomposites. As shown in Figure 7d, Young’s modulus for MAPOSS composite increases with an increase in the corresponding weight % of MAPOSS up to 5 wt% and then it decreases. But it decreases in MIPOSS with an increase in the corresponding weight % of MIPOSS. Since the observed degree of conversion in both MAPOSS and MIPOSS is the same and over 95%, it can be concluded that the variation in Young’s modulus is not due to conversion. Emami and Soderholm [24] also concluded in their experimental study on the effect of conversion on Young’s modulus of light cure dental resins made of BisGMA, TEGDMA, and diurethane dimethacrylate. It is the molecular structure of the POSS along with the monomers and its effect on the polymer network that has a significant influence on Young’s modulus. Since MIPOSS is not molecularly bound to the chains and forms aggregates, it tends to lower the elasticity while MAPOSS bounds molecularly to the polymer chains, increasing the elasticity. With only POSS as an additive to polystyrene, the rubbery elastic modulus was decreased similar to MIPOSS composites as reported by Romo-Uribe [25] though the main reason in that study was found to be the disentanglement of chains that caused the decrease in the Young’s modulus. On the other hand, Chatterjee et al. [26] experimentally showed that MAPOSS additive increased the Young’s modulus in a biocomposite made of methacrylate-POSS and 2-(methacryloyloxy)ethyl phosphorylcholine. Wang et al. [27] showed that the modulus increases with an increase in the weight percent of POSS in BisGMA and TGDMA up to 5 weight % and then it decreases with further addition of POSS to the composite, which is quite similar to the results obtained in our study. While both MAPOSS and MIPOSS composites are used for making dental composites, based on the simulation results obtained in this study which comprehends the experimental findings of Wang et al. [9] it can be concluded that MAPOSS composite has more benefits compared to MIPOSS composite.

## 4. Conclusions

Nanocomposites of BisGMA, TEGDMA, MAPOSS, and MIPOSS were analyzed using molecular dynamics simulations. The structure interaction of MAPOSS and MIPOSS was confirmed and complemented by the experimental findings reported by Wang et al. [9]. It was observed that the density of MAPOSS composite increased with an increase in the weight percent of MAPOSS added while the density decreased with an increase in the weight percent of MIPOSS in the composite. The Young’s modulus calculated within the elastic limit of the stress–strain results obtained for all the different composites showed that it increases with the MAPOSS up to 5 wt% and then decreases while it continuously decreases with an increase in the wt% of MIPOSS. It was found that the double bonds in MAPOSS participate in the crosslinking of the polymers, thereby becoming part of the polymer matrix. On the other hand, MIPOSS molecules were present as stand-alone in the polymer matrix and formed aggregates of varying sizes which led to the deterioration of properties compared to the Control and also MAPOSS composites. These findings were confirmed from the density projection and powder diffraction analysis, which were also complemented by the experimental results reported in the available literature. The results obtained from this study can be successfully used to design, build, and study new composites using computational simulations, reducing the cost of performing experiments and saving experimentation time.

## Figures and Tables

**Table 1:** Composition and individual number of molecules of different composites.

Composite	BisGMA (wt%)	TEGDMA (wt%)	POSS (wt%)	BisGMA (Number of Molecules)	TEGDMA (Number of Molecules)	POSS (Number of Molecules)
Control	50	50	0	299	381	0
POSSMA1	49.5	49.5	1.0	207	372	2
POSSMA3	48.4	48.5	3.3	204	364	5
POSSMA5	47.5	47.4	5.3	199	357	8
POSSMA10	45.0	45.0	10.0	189	338	15
POSSMI1	49.4	49.4	1.2	211	378	3
POSSMI3	48.4	48.4	3.2	207	370	8
POSSMI5	47.4	47.4	5.2	202	362	13
POSSMI10	45	45	10	192	343	25

## Data Availability

We provided all information related to the simulations and data in the article. For any additional information they can contact the corresponding author.
